# CRISPR‐Cas9‐mediated efficient directed mutagenesis and RAD51‐dependent and RAD51‐independent gene targeting in the moss *Physcomitrella patens*

**DOI:** 10.1111/pbi.12596

**Published:** 2016-07-22

**Authors:** Cécile Collonnier, Aline Epert, Kostlend Mara, François Maclot, Anouchka Guyon‐Debast, Florence Charlot, Charles White, Didier G. Schaefer, Fabien Nogué

**Affiliations:** ^1^ INRA Centre de Versailles‐Grignon IJPB (UMR1318) Versailles Cedex France; ^2^ Génétique, Reproduction et Développement UMR CNRS 6293 Clermont Université INSERM U1103 Université Blaise Pascal Clermont Ferrand France; ^3^ Laboratoire de Biologie Moléculaire et Cellulaire Institut de Biologie Université de Neuchâtel Neuchâtel Switzerland

**Keywords:** CRISPR‐Cas9, *Physcomitrella patens*, genome editing, alt‐EJ, gene targeting, RAD51

## Abstract

The ability to address the CRISPR‐Cas9 nuclease complex to any target DNA using customizable single‐guide RNAs has now permitted genome engineering in many species. Here, we report its first successful use in a nonvascular plant, the moss *Physcomitrella patens*. Single‐guide RNAs (sgRNAs) were designed to target an endogenous reporter gene, *PpAPT,* whose inactivation confers resistance to 2‐fluoroadenine. Transformation of moss protoplasts with these sgRNAs and the Cas9 coding sequence from *Streptococcus pyogenes* triggered mutagenesis at the *PpAPT* target in about 2% of the regenerated plants. Mainly, deletions were observed, most of them resulting from alternative end‐joining (alt‐EJ)‐driven repair. We further demonstrate that, in the presence of a donor DNA sharing sequence homology with the *PpAPT* gene, most transgene integration events occur by homology‐driven repair (HDR) at the target locus but also that Cas9‐induced double‐strand breaks are repaired with almost equal frequencies by mutagenic illegitimate recombination. Finally, we establish that a significant fraction of HDR‐mediated gene targeting events (30%) is still possible in the absence of PpRAD51 protein, indicating that CRISPR‐induced HDR is only partially mediated by the classical homologous recombination pathway.

## Introduction

Over the last decades, gene editing and transgene integration have been shown to be facilitated by the generation of a DNA double‐strand break (DSB) at targeted genomic locations, using homing endonucleases such as meganucleases, zinc finger nucleases (ZFNs) and TAL effector nucleases (TALENs). Recently, a new type of site‐directed nucleases based on the prokaryotic type II CRISPR‐Cas9 (clustered regularly interspaced short palindromic repeats/CRISPR‐associated protein) immune system has been used for precise genome editing in many species with spectacular success (Lander, 2016). In this system, a Cas9 endonuclease protein from *Streptococcus pyogenes* guided by a customizable noncoding RNA introduces site‐specific double‐strand DNA breaks (DSBs) in the genome. Repair of these DSBs can lead to gene disruption if the break is repaired by a deleterious event resulting from a classical nonhomologous end‐joining reaction (C‐NHEJ), an alternative end‐joining reaction (alt‐EJ, also called microhomology‐mediated end joining, MMEJ) or a single‐strand annealing reaction (SSA; Ceccaldi *et al*., 2016). Alternatively, in the presence of a homologous donor DNA template, these DSBs can be repaired via a homology‐directed repair (HDR) pathway, leading to accurate gene replacement (Ceccaldi *et al*., 2016). The feasibility of such approach has been demonstrated in several eukaryotic cells and raises great expectations for gene therapeutic approaches. However, highly efficient HDR of CRISPR‐Cas9‐induced genomic DSB remains so far restricted to GT‐competent cells such as budding yeast (Dicarlo *et al*., 2013).

In plants, the CRISPR‐Cas9 system has been applied with good efficiency for the induction of illegitimate recombination‐mediated (IR) targeted mutagenesis—or knockout—of endogenous loci (Schaeffer and Nakata, 2015). It constitutes a revolutionary tool for functional gene analysis, but also a promising approach for the development of new traits of interest in crops (Collonnier *et al*., 2016; Petolino *et al*., 2016). By comparison, the examples of integration of a donor DNA template sharing homology with the target and leading to gene replacement through HDR‐based repair, namely gene targeting (GT), are rare, hardly reaching the percentage range (Čermák *et al*., 2015; D'Halluin *et al*., 2013; Li *et al*., 2013; Nishizawa‐Yokoi *et al*., 2015; Schiml *et al*., 2014; Shukla *et al*., 2009; Townsend *et al*., 2009). This reflects the fact that very low GT efficiency is generally observed in higher plants, reaching only 0.01% to 0.1% of the effectively transformed plants (Hanin and Paszkowski, 2003). Even if site‐directed nucleases, such as the CRISPR‐Cas9 system, help for the targeted modification of genes, this strategy is still challenging compared to gene knockout (Schiml and Puchta, 2016). High GT frequencies are naturally achieved only by a few species/cell lines (Schaefer, 2001), and this is thought to be associated with the fact that homologous recombination (HR) is the principal mechanism for DSBs repair, as exemplified in *Saccharomyces cerevisiae* (Pâques and Haber, 1999). The moss *Physcomitrella patens* is the only plant that naturally displays high GT efficiencies (Schaefer and Zrÿd, 1997), and recent genetic studies have shown that this feature is tightly associated with the classical RAD51‐mediated HR repair pathway (Charlot *et al*., 2014; Kamisugi *et al*., 2012; Schaefer *et al*., 2010). Efficient GT, the availability of a completely sequenced genome and unique genetic and developmental facilities have established *P. patens* as a valuable novel model system in plant biology (Bonhomme *et al*., 2013; Kofuji and Hasebe, 2014; Prigge and Bezanilla, 2010).

Here, we report the first successful use of the CRISPR‐Cas9 system to achieve both targeted mutagenesis and gene targeting in *Physcomitrella patens*. To monitor the RNA‐guided Cas9 nuclease activity, we designed sgRNAs targeting the endogenous adenine phosphoribosyltransferase (*PpAPT*) selectable marker gene whose loss of function confers resistance to 2‐fluoroadenine (2‐FA; Charlot *et al*., 2014; Kamisugi *et al*., 2012; Schaefer *et al*., 2010; Trouiller *et al*., 2006, 2007). We show that PEG‐mediated transformation of moss protoplasts with these sgRNAs and the Cas9 coding sequence from *Streptococcus pyogenes* efficiently induces targeted mutagenesis of the *PpAPT* gene in 2% to 3% of the regenerated plants. Molecular analyses revealed that these mutations result from a diversity of deletions, insertions and/or substitutions in the *PpAPT* locus, confirming the efficiency of the CRISPR‐Cas9 system for gene knockout/editing in *P. patens*.

To evaluate the impact of CRISPR/Cas9 on GT, we performed moss transformation with the sgRNA sequences, the Cas9 gene and a circular donor plasmid bearing an antibiotic resistance gene flanked by DNA fragments homologous to the genomic regions flanking the target. Our analyses reveal that HDR‐mediated integration of the donor DNA in the *PpAPT* locus occurs in almost 100% of the transformed plants (i.e. proportion of 2‐FA^R^ among antibiotic‐resistant plants). Molecular analyses further indicate that the proportion of single‐copy replacements is significantly increased compared to the classical approach with linearized replacement vectors. Interestingly, our data also demonstrate that approximately 40% (i.e. proportion of antibiotic resistant among 2‐FA^R^ plants) of Cas9‐induced DSBs are not repaired by HDR in this situation, indicating that a significant fraction of these DSBs are repaired by mutagenic IR or end‐joining reactions.

Finally, we assessed CRISPR‐Cas9‐mediated GT efficiency in the *Pprad51‐1‐2* double mutant, as we previously established that this gene was essential to achieve GT using linearized replacement vectors (Schaefer *et al*., 2010; Wendeler *et al*., 2015). Unexpectedly, this analysis revealed that HDR‐mediated GT was reduced but not abolished in the mutant, reaching approximately 30% of the WT level. This observation implies that other types of DNA repair pathways are involved in the integration of the donor template when Cas9 generates a DSB at the chromosomal target gene. Thus, the use of the CRISPR‐Cas9 system significantly improves GT efficiency and precision in *P. patens*, expanding the range of available tools for gene function analysis in this model organism. These data also uncover novel features of CRISPR‐induced HDR‐mediated GT that could lead to improve the efficiency of such approach in GT noncompetent cells.

## Results

### Highly efficient gene knockout in *P. patens* with RNA‐guided Cas9 nuclease

**Figure 1:**
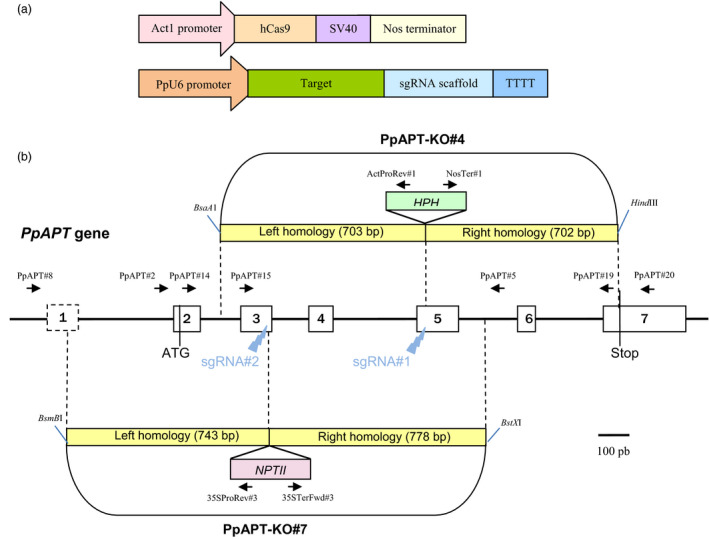
Schematic description of the sgRNA/Sp‐hCas9 system and of the PpAPT reporter gene. (a) Design of the pAct‐Cas9 and psgRNAs constructs. (b) Structure of PpAPT with the target sites and the homologies of the donor cassette (PpAPT‐KO4 and PpAPT‐KO7) used for gene targeting experiment (white rectangles represent exons). For classical GT experiments, donor DNA was released using restriction enzymes as indicated.

**Figure 2:**
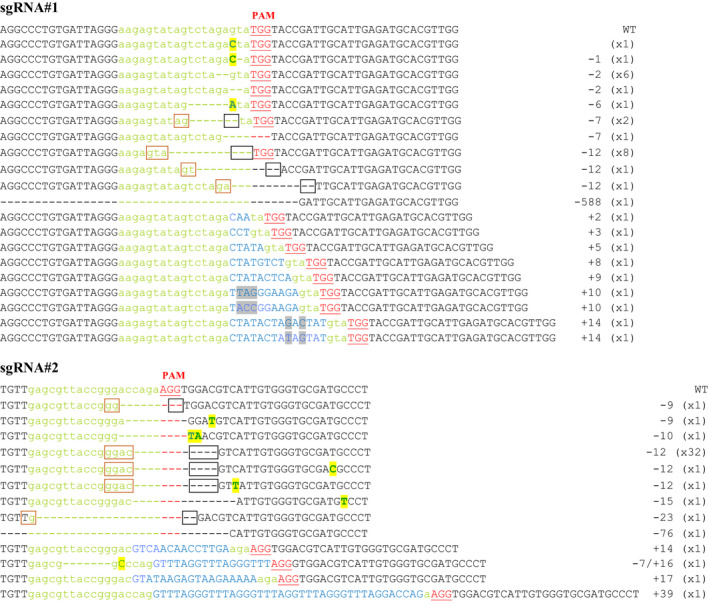
Targeted genome editing on *PpAPT* gene in *Physcomitrella patens* protoplasts. Green letters indicate the sequences targeted by the tested sgRNAs. DNA insertions are shown in blue, point mutations in bold capital letters on a yellow background and deletions with dashes. With a grey background, the differences between two insertions of the same length. The PAM (protospacer adjacent motif) is marked in red underlined letters. In the frames, the microhomologies potentially involved in alt‐EJ‐mediated repair of the CRISPR‐induced DSBs. For each deletion potentially due to alt‐EJ, the brown frame surrounds the 5′ homology, and the black one, the position of the 3′ homology before alt‐EJ occurred and produced the deletion represented by dashes (see Figure S3).

To evaluate the potential of the CRISPR‐Cas9 system to induce targeted mutagenesis in *P. patens,* two sgRNAs matching, respectively, two target loci in exon 3 (sgRNA#2) and exon 5 (sgRNA#1) of the *PpAPT* reporter gene were designed (Figures 1 and S1). *P. patens* wild‐type protoplasts were cotransformed by PEG‐mediated transformation with two plasmids, one bearing the Cas9 gene under the control of the rice actin 1 promoter (pAct‐Cas9) and another bearing the sgRNA#1 or sgRNA#2, both under the control of a *P. patens* U6 promoter. Mutations leading to a loss of APT activity confer resistance to toxic adenine analogues, such as 2‐fluoroadenine (2‐FA; Schaff, 1994; Trouiller *et al*., 2006). The mutation rates (expressed in percentages) were estimated by dividing the number of 2‐FA‐resistant plants by the number of regenerating plants observed just before the transfer on 2‐FA. The mutation rates obtained using sgRNA#1 and sgRNA#2 were, respectively, 2.2% and 3.2% (Table 1) and were optimal when the sgRNAs and Cas9 plasmids were provided in a 1 : 1 ratio (Figure S2). To characterize these mutations, we amplified by PCR and sequenced the *PpAPT* gene in 34 independent clones for sgRNA#1 and 43 independent clones for sgRNA#2 (Figure 2). As expected, all the mutations were located in the vicinity of the PAM target of the Cas9‐induced cleavage site (Gasiunas *et al*., 2012) and generated loss of APT function. These mutations consisted of deletions of 1 to 588 bp, insertions of 2 to 39 bp and substitutions of 1 to 2 bp, a majority of the mutations being deletions (Table 2). Generally, the substitutions occurred inside the target sequence but they were also observed up to 22 bp downstream of the PAM. Regarding the insertions, they all occurred a few nucleotides upstream of the PAM and for sgRNA#1, 8 of 9 occurred exactly at the same position 3 bp before the PAM. Interestingly, for a large number of the deletions (12/23 simple deletion events for sgRNA#1 and 36/40 for sgRNA#2), microhomologies (of 2 to 4 bp) could be detected between the end of the deletion itself and the sequence located just upstream of the deletion (Figure 2). With sgRNA#1, 8 events could be explained by alt‐EJ‐mediated repair based on 3‐bp‐long microhomologies and 4 events on 2‐bp‐long microhomologies. With sgRNA#2, 34 events could be explained by alt‐EJ‐mediated repair based on 4‐bp‐long microhomologies and 2 events on 2‐bp‐long microhomologies (Figure 2). Thus, the CRISPR‐Cas9 system is very efficient to induce targeted mutagenesis in *P. patens*, and the repair of an induced genomic DSB seems to implicate both C‐NHEJ and alt‐EJ mechanisms (Figure S3).

**Table 1:** *PpAPT* gene knockout efficiency using the CRISPR‐Cas9 system

sgRNA used for transformation^a^	Regenerant clones	2‐FA^R^ clones	Knockout efficiency (%)
No sgRNA	15 000	0	0
sgRNA#1	149 175 (49 725 ± 3857^b^)	3321 (1107 ± 107^b^)	2.2 ± 0.09^b^
sgRNA#2	164 650 (54 883 ± 2819^b^)	5229 (1743 ± 140^b^)	3.2 ± 0.16^b^

a sgRNA#1 and sgRNA#2 target exon 5 and exon 3 of the *PpAPT* gene respectively (see Figure 1b).

b Average and standard deviations were determined from three independent experiments.

**Table 2:** Types of CRISPR‐Cas9‐induced mutations

sgRNA used for transformation^a^	Number of analysed clones	No. of clones with deletions (%)	No. of clones with insertions (%)	No. of clones with substitutions (%)
sgRNA#1	34	23 (67)	10 (29)	3 (9)
sgRNA#2	43	40 (93)	4 (9)	5 (9)

a sgRNA#1 and sgRNA#2 target exon 5 and exon 3 of the *PpAPT* gene respectively (see Figure 1b).

### RNA‐guided nuclease activity is very specific in *P. patens*

The sgRNAs used in this study were designed to minimize potential off‐target cleavage in the *P. patens* genome (Phytozome 3.1) using the CRISPOR software. For both the selected targets, no perfect 20‐bp matches were found but potential off‐target sequences presenting 3 to 6 mismatches were identified: 9 for sgRNA#1 and 3 for sgRNA#2 (Figure S4). All these potential off‐target loci were amplified with surrounding primers (Figure S5) and sequenced in 48 clones transformed with hCas9 and sgRNA#1 and 48 clones transformed with hCas9 and sgRNA#2 that were all previously identified as mutated at the *PpAPT* locus. No mutation could be detected in the potential off‐target sequences for any of the tested clones.

### CRISPR‐Cas9 system increases gene targeting efficiency and single‐copy replacement in *P. patens*

The impact of the CRISPR‐Cas9 system on gene targeting efficiency was evaluated by cotransferring into protoplasts three circular plasmids, one expressing the Cas9 gene (pAct‐Cas9), one expressing the sgRNA#2 and one carrying the donor cassette PpAPT‐KO7 which bears a G418‐resistant gene surrounded by *PpAPT* gene sequences flanking the sgRNA target (Figure 1b). In parallel, we performed a classical gene targeting experiment and transformed protoplasts with the linearized replacement cassette PpAPT‐KO7 (Figure 1b). In classical GT experiments, HR‐mediated integration of a replacement vector can occur in 2 distinct ways: by targeted gene replacement (TGR) or by targeted gene insertion (TGI). TGR is mediated by two HR reactions involving both homologous sequences of the vector and leads to accurate gene replacement. TGI results from a single HR involving one of the homologous sequence of the vector on one side of the target, associated with an IR event involving the other homologous sequence on the other side (Kamisugi *et al*., 2006; Schaefer, 2002; Schaefer and Zrÿd, 2004). To evaluate the efficiency of gene targeting through HDR, regenerating moss plants from the ‘CRISPR‐Cas9’ or ‘classical’ transformations were sequentially subcultured first on media containing the antibiotic G418 and then on 2‐FA. Our analysis showed that relative transformation frequency (RTF: i.e. frequency of G‐418^R^ plants) was much higher using the ‘CRISPR‐Cas9’‐mediated transformation (2.1%) compared to the ‘classical’ transformation method (0.25%, Table 3). It further demonstrated that GT efficiencies (i.e. the percentage of 2‐FA‐resistant plants among G418‐resistant plants) are significantly increased, reaching 100% following CRISPR‐mediated transformation compared to 54% with the classical strategy (Table 3, Fisher's exact test *P* = 0.008).

**Table 3:** Comparison of *PpAPT* gene targeting efficiency using the ‘CRISPR‐Cas9’ versus ‘classical’ mediated transformations

Type of transformation^a^	RTF^b^ %	AB^R^ clones	2‐FA^R^ clones^c^	Integration due to IR^d^	Integration due to HDR^e^		GT^f^ %
					TGR	TGI (5′TGI + 3′TGI)	
‘CRISPR‐Cas9’	2.1 ± 0.2	95	95	0	84	11 (7 + 4)	100
‘Classical’	0.25 ± 0.1	95	52	43	40	12 (7 + 5)	54.7

a For ‘CRISPR‐Cas9’‐mediated transformation, wild‐type protoplasts were cotransformed with pAct‐Cas9, psgRNA#2 and circular PpAPT‐KO7 donor DNA cassette. For ‘classical’ mediated transformation, wild‐type protoplasts were transformed with linear PpAPT‐KO7 donor DNA cassette.

b Relative transformation frequencies (RTF) express the frequency of stable AB^R^ clones in the population of regenerated clones. A total of 2580 and 123 AB^R^ clones were obtained for the ‘CRISPR‐Cas9’ and ‘classical’ methods of transformation, respectively. Standard deviation was determined from 3 independent experiments.

c 2‐FA^R^ clones are the stable AB^R^ clones that survived after subculture on 2‐FA medium. They all result from a homology‐driven recombination (HDR) event (see Figure S6).

d Number of AB^R^ clones where the donor DNA template has been randomly inserted by illegitimate recombination (IR) and not via HDR.

e Number of 2‐FA^R^ clones resulting from HDR (TGR or TGI)‐mediated insertion of the donor DNA template at the *PpAPT* locus was determined by PCR analysis (see Figure S6). Clones resulting from TGR show 5′ and 3′ junction, clones resulting from 5′ TGI show only a 5′ junction, and clones resulting from 3′ TGI show only a 3′ junction.

f GT efficiencies (%) express the frequency of 2‐FA‐resistant clones among the population of antibiotic‐resistant transgenic clones.

At the molecular level, junction analyses by PCR genotyping of the *PpAPT* locus in G418 and 2‐FA‐resistant plants (*n* = 95 for the ‘CRISPR‐Cas9’‐mediated transformation and *n* = 52 for the ‘classical’ mediated transformation) provided evidence for the integration of the donor vector by at least one HR event in all the plants isolated from both procedures, leading to either TGR or TGI events (Figure S6). The ratio between the two types of events is not significantly different between the two methods of transformation (Table 3).

Thus, our analyses revealed that the use of CRISPR‐Cas9 increases both transformation frequencies (8.4‐fold) and GT efficiencies (1.8‐fold). These results also show that when a DSB is induced at the chromosomal target site, the linearization of the donor DNA template is no longer a prerequisite for efficient GT. Finally, as observed before for classical mediated transformation, ‘CRISPR‐Cas9’‐mediated transformation can lead to TGR or TGI of the donor DNA template.

**Figure 3:**
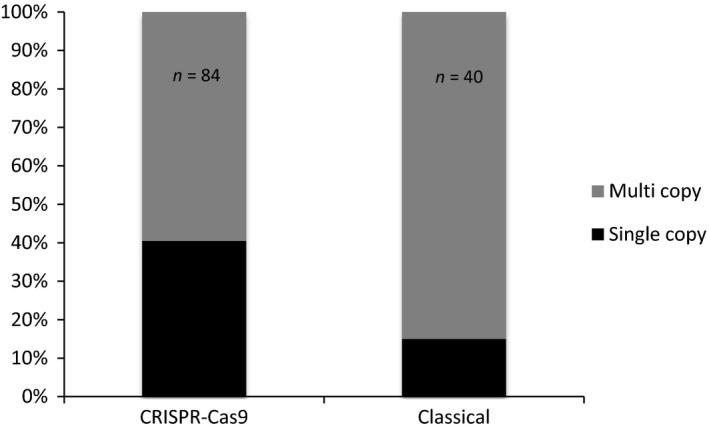
Ratio of single copy versus multiple copies of donor DNA template insertions using the ‘CRISPR‐Cas9’ or the ‘classical’ methods of transformation in *Physcomitrella patens*. Frequency of single‐copy TGR insertions was determined by genotyping the 2‐FA^R^ clones (84 for the ‘CRISPR‐Cas9’ method and 40 for the ‘classical’ method, see Table 3) using primers located outside the sequences homologous to the gene fragments present in the PpAPT‐KO7 donor DNA template (see Figure S7).

Insertion of concatenated copies of the donor cassettes is frequent in GT experiments in *P. patens* (Kamisugi *et al*., 2006; Schaefer and Zrÿd, 1997). We further genotyped these plants to detect TGR events in which a single copy of the donor cassette had been integrated at the target site, using genomic primers located upstream and downstream of the *PpAPT* sequences present in PpAPT‐KO7 (Figure S7). This analysis showed that the number of clones carrying only one copy of the cassette was significantly higher (Fisher's exact test *P* = 0.04) with the ‘CRISPR‐Cas9’‐mediated transformation (40.5%) than with the ‘classical’ method (15%; Figure 3). This significant increase in single‐copy insertions at the *APT* locus using the CRISPR‐Cas9 strategy has to be confirmed for others loci in *P. patens*. The use of CRISPR‐Cas9 could possibly reduce the problem of insertion of concatemers at the target site, a frequent occurrence in *P. patens* transformation. Altogether, our results show that the induction of a chromosomal DSB at the target site increases the frequency, the efficiency and the accuracy of GT in *P. patens*, compared to the classical approach with linearized replacement vectors.

### In the presence of a donor template, CRISPR/Cas9‐induced DSBs are repaired either by HDR or by IR

As shown above, integration of a donor DNA template occurs almost exclusively by HDR when a DSB is produced by the CRISPR‐Cas9 system at the chromosomal target (Table 3, 95 of 95 for sgRNA#2/PpAPT‐KO7). We also showed that, in absence of a donor DNA template, Cas9‐induced DSBs can be efficiently repaired via C‐NHEJ or alt‐EJ, leading to deletions, substitutions or insertions (Table 2). To evaluate the proportions of these two types of potentially concurrent events, we transformed moss with the Cas9 cassette, the sgRNA#2 and the donor DNA cassette PpAPT‐KO7 and selected the plants initially for 2‐FA resistance and then for G418 resistance among 2‐FA^R^ plants. In this situation, we isolated approximately 2 times more 2FA^R^ plants (4%, Table 4) than upon initial selection for antibiotic resistance (2.1%, Table 3). Consistently, this experiment revealed that only 60% of CRISPR‐Cas9‐induced DSBs are repaired by HDR‐mediated targeted integration of the donor DNA template (121/200), while the remaining 40% are repaired by IR events (C‐NHEJ or alt‐EJ; Table 4). This indicates clearly that both homology‐driven and illegitimate recombination pathways are equally proficient to repair Cas9‐induced chromosomal DSBs in *P. patens* even in the presence of a homologous donor template.

**Table 4:** Proportion of HDR‐ versus IR‐mediated repair of CRISPR‐Cas9‐induced DSB at the *PpAPT* locus, in the presence of a donor cassette

	Number of 2‐FA^R^ clones analysed^a^	AB^R^ clones from 2‐FA^R^ clones^b^	HDR‐mediated DSB repair %^c^	IR‐mediated DSB repair %^d^
pAct‐Cas9 + psgRNA#2 + PpAPT‐KO7	200	121	60	40

a A total of 3750 2FA^R^ clones were obtained from 3 independent experiments.

b AB^R^ clones are the stable 2‐FA^R^ clones that survived after subculture on G418 medium.

c HDR‐mediated DSB repair (%) expresses the frequency of G418‐resistant clones among the population of 2‐FA^R^‐resistant clones. HDR‐mediated repair of the G418^R^ clones was confirmed by PCR analysis of the *PpAPT* locus (not shown, same as Figure S6).

d IR‐mediated DSB repair (%) expresses the frequency of G418‐sensitive clones among the population of 2‐FA^R^‐resistant clones.

### CRISPR‐Cas9‐induced targeted integration in *P. patens* is only partially RAD51 dependent

The RAD51 protein catalyses most homologous recombination reactions in eukaryotes and is directly involved in homology searching, homologous pairing and DNA strand transfer (Holthausen *et al*., 2010). Previous studies have shown that gene targeting in *P. patens* was totally abolished in the absence of PpRAD51‐1 and PpRAD51‐2 proteins (Schaefer *et al*., 2010). To determine whether CRISPR‐induced HDR‐mediated targeted integration of a donor DNA template depends on the same HR DNA repair pathway, CRISPR‐induced GT was analysed in the double‐mutant *Pprad51‐1‐2* (Schaefer *et al*., 2010). We transformed wild‐type and *Pprad51‐1‐2* protoplasts with the Cas9 cassette, the sgRNA#1 and the donor DNA cassette PpAPT‐KO4, bearing a hygromycin‐resistant gene surrounded by *PpAPT* gene sequences flanking the sgRNA target (Figure 1b). Regenerated plants were then sequentially selected for hygromycin resistance first, and then for resistance to 2‐FA. RTFs were not significantly different between the two strains and similar to those observed in the previous CRISPR‐Cas9 experiment, indicating that both genotypes are equally competent for transformation (Table 5). In the wild‐type strain, 94% (47/50) of the hygromycin‐resistant clones were also 2FA^R^ (Table 5). At the molecular level, PCR genotyping provided evidence for the presence of junctions generated by HR and for the successful disruption of the *PpAPT* locus in 46 of them, supporting HDR‐mediated targeted integration (TGR or TGI) of the donor DNA template (Table 5 and Figure S8). The last plant did not show junctions corresponding to a HR event but shows an interruption of the *PpAPT* gene. This is consistent with the integration of PpAPT‐KO4 through illegitimate recombination in the DSB generated by Cas9 at the *PpAPT* locus. Thus, GT efficiency in the wild type reached 94% (47/50) using the sgRNA#1/PpAPT‐KO4 donor cassette couple (Table 5), which is similar to the efficiency observed with the sgRNA#2/ PpAPT‐KO7 couple (Table 3) and confirms that the ‘CRISPR‐Cas9’‐mediated GT is remarkably efficient in moss.

**Table 5:** Comparison of ‘CRISPR‐Cas9’‐mediated *PpAPT* gene targeting efficiency in wild‐type and the double‐mutant *Pprad51‐1‐2*

	AB^R^ clones^a^	2‐FA^R^ clones^b^	Integration due to HDR^c^		Integration due to IR		GT^e^ %
			TGR	TGI (5′TGI + 3′TGI)	*PpAPT* locus^c^	Random^d^	
Wild type	50	47	39	7 (3 + 4)	1	0	94
*Pprad51‐1‐2*	102	93	3	26 (7 + 19)	34	30	91

a From 2 independent experiments, a total of 1680 and 1800 AB^R^ clones were obtained for the wild‐type and the *Pprad51‐1‐2* mutant, respectively.

b 2‐FA^R^ clones are the stable AB^R^ clones that survived after subculture on 2‐FA medium.

c Number of 2‐FA^R^ clones resulting from HDR‐ (TGR or TGI) or IR‐mediated insertion of the donor DNA template at the *PpAPT* locus was determined by PCR analysis (see Figure S8). Clones resulting from TGR show 5′ and 3′ junction, clones resulting from 5′ TGI show only a 5′ junction, and clones resulting from 3′ TGI show only a 3′ junction.

d Number of 2‐FA^R^ clones where the donor DNA template has been randomly inserted by IR was determined by PCR analysis (see Figure S8).

e GT efficiencies (%) express here the frequency of 2‐FA^R^ clones resulting from HDR‐mediated targeted insertion among the population of antibiotic‐resistant transgenic clones.

**Figure 4:**
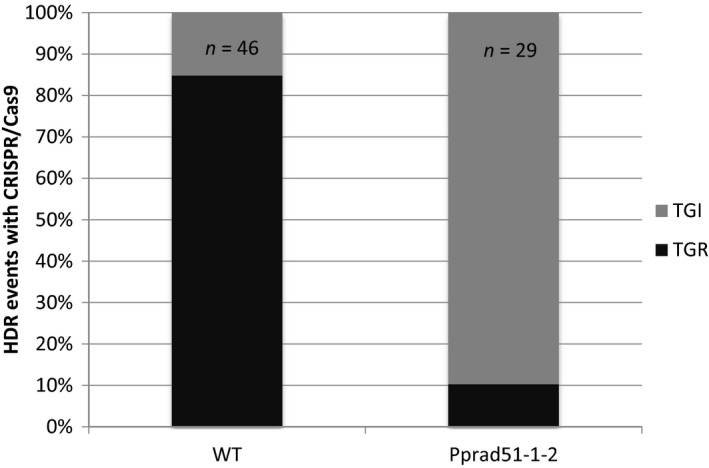
Ratio of TGR versus TGI insertions of the donor DNA template in the wild‐type and *Pprad51‐1‐2* mutant. Frequency of TGR and TGI insertions was determined by genotyping the 2‐FA^R^ clones resulting from HDR (46 for the wild‐type and 29 for the *Pprad51‐1‐2* mutant, see Table 4) using primers specific to the PpAPT‐KO4 cassette and primers located on the *PpAPT* gene but outside of the genomic fragments present on the donor DNA cassette (see Figure S8).

For the *Pprad51‐1‐2* mutant, the percentage of 2‐FA^R^ plants among hygromycin‐resistant plants reached 91% (93/102), which was similar to what was observed in the wild type (Table 5). However, PCR genotyping of the *PpAPT* locus and of the recombined junctions identified 3 distinct classes of transformed plants (Table 5 and Figure S8). Disruption of the *PpAPT* locus associated with the generation of at least one of the junctions *via* HR was observed in 29 of them, providing evidence for HDR‐mediated targeted integration of the vector at the *PpAPT* locus. In 30 of them, the *PpAPT* locus appeared intact and no junctions could be detected, which is consistent with a random integration of the donor DNA template in the genome accompanied by the repair of the CRISPR‐Cas9‐induced DSB in the *PpAPT* gene through C‐NHEJ or alt‐EJ pathways. This was further confirmed by sequence analysis of the PCR product (data not shown). In the last 34 plants, PCR data indicated that the *PpAPT* locus was disrupted without the generation of predicted recombined junctions. Such pattern is consistent with the integration of the donor vector by IR in the DSBs generated by Cas9 at the *PpAPT* locus as previously observed in one WT plant described above. Finally, junction analysis of HDR‐mediated events also revealed a significant change of the TGR/TGI ratio: TGR events occurred in 85% (39/46 HDR events) of the WT, as previously observed with sgRNA#2 (88%, Table 3), but only in 10.3% (3/29 HDR events) of the *Pprad51‐1‐2‐*transformed plants (Figure 4, Fisher's exact test *P* = 1.8 × 10^−4^).

These data show that in the *Pprad51‐1‐2* double‐mutant, HDR‐mediated targeted integration of the donor DNA template reached 28.4% (29/102) and is decreased by threefold compared to the wild type [92% (46/50) to 28.4%], while integration by illegitimate recombination at random loci or at Cas9‐generated DSB is dramatically increased [2% (1/50) in the WT *versus* 63% (64/102) in the mutant]. Furthermore, the residual HDR‐mediated targeted integrations found in the mutant consist mostly of TGI events resulting from a single HR reaction within one of the homologous sequence of the donor DNA template (confirmed by sequencing analysis, data not shown), accompanied by an apparent NHEJ reaction at the other end of the cassette. The RAD51 function being essential for DNA repair *via* homologous recombination, the residual HDR events found in the *Pprad51‐1‐2* mutant context are probably due to other homology‐driven DNA repair mechanisms.

## Discussion

In the present study, we set up and demonstrate for the first time in a bryophyte, *Physcomitrella patens,* the potential of the CRISPR‐Cas9 system to induce targeted mutagenesis in the genome. We obtained fully developed 2‐FA^R^ plants at very high frequencies (2.2% to 3.2%) and harbouring a large variety of mutations including deletions, substitutions and insertions at the *PpAPT* target gene. The deletions we observed could result from two repair pathways, either from C‐NHEJ or from alt‐EJ, this last pathway relying on 2‐ to 4‐bp microhomology regions (Figure S3). One way to check this hypothesis will be to perform the same experiments in mutants impacted in these DNA repair pathways. The types of mutations we obtained are consistent with those reported in different plant species where the CRISPR‐Cas9 system has been used so far, and that describes mainly small deletions (usually <20 bp), small insertions of a few bp and rare single‐nucleotide substitutions.

Off‐targeting is a concern for CRISPR‐Cas9‐induced mutagenesis in human cells (Fu *et al*., 2013), and new Cas9 proteins with enhanced specificity have been engineered recently (Kleinstiver *et al*., 2015; Slaymaker *et al*., 2015). In mice and zebrafish, lower off‐target activities have been described compared to human cells (Hruscha *et al*., 2013; Wang *et al*., 2013). In plants, no or very low off‐targeting activity has been reported (Feng *et al*., 2014; Xie and Yang, 2013). Potential off‐targeting of the sgRNAs used in this study was evaluated by searching the *P. patens* genome for other genomic occurrences of the selected target sequences with a tolerance of a few mismatches. After sequencing of those targets, no off‐targeting could be detected. A whole‐genome deep sequencing could be more extensive.

We then compared GT efficiencies and monitored targeted integration of a donor plasmid following CRISPR‐Cas9‐mediated generation of a DSB in the *PpAPT* locus *versus* classical transfection with a linear *PpAPT* replacement vector. We consider that the major difference between these two situations resides in the fact that the cells have to deal with the repair of a single DSB in the former one, and with a massive signal of DSB damage in the latter. Our data first show that the integration of a transgene is 10‐fold more frequent when a single chromosomal DSB is generated by the CRISPR‐Cas9 system compared to the delivery of linearized vectors (cf. relative transformation frequencies in Table 3). They also indicate that the cellular competency for transgene integration through HDR is significantly increased when the moss cell has to repair a single chromosomal break, as previously reported in many other systems (Puchta, 2005). Our data further demonstrate that GT efficiency (i.e. % of 2‐FA^R^ in AB^R^ plants in Table 3) reaches almost 100% following CRISPR transformation, which slightly but significantly (Fisher's exact test: *P* = 0.008) improves the naturally high GT efficiencies of *P. patens* (Tables 3 and 5). They also show that the pattern of GT is not significantly different and that Cas9‐induced HDR leads to either TGR or TGI events as observed in classical transformation. This indicates that transgene integration occurs almost always in a Cas9‐generated DSB and that the cells use a similar combination of HR and IR reactions to repair this DSB. Our analysis also revealed a significant decrease in the frequency of tandem repeat integration of the donor DNA after HDR‐mediated GT (Figure 3), which is probably associated with the fact that the donor DNA is provided as a circular molecule. Finally, we show that only 60% of Cas9‐induced DSBs are repaired by HDR when selection is performed for loss of PpAPT function prior to donor DNA integration (Table 4). This clearly demonstrates that Cas9‐induced DSB are repaired with almost equal efficiencies by HR or IR in moss and is in sharp contrast with the general idea that efficient GT correlates with the dominance of the HR pathway in DSB repair.

Altogether, this analysis shows that the use of CRISPR/Cas9 significantly improves the naturally high capacities to perform accurate modifications of the moss genome, with GT efficiencies reaching 100%. Such efficiencies have only been reported so far for *S. cerevisiae* (Dicarlo *et al*., 2013) and clearly demonstrate that the competency for GT is essential to achieve efficient CRISPR‐Cas9 HDR‐mediated gene replacement, meaning that in nonproficient GT organisms, such as vascular plants for example, if CRISPR‐Cas9 can help HDR‐mediated gene replacement, it is not sufficient to use this strategy in routine. Our results show that the competency for GT found in *P. patens* is not due to the Cas9‐induced DSB being essentially repaired through the HR pathway as both IR and HR equally contribute to these events. Further work will be needed to decipher the mechanism controlling the choice for DSB repair pathways in *P. patens*, and to elucidate the respective contributions of C‐NHEJ and alt‐EJ in the IR repair pathway.

GT in *P. patens* using linearized replacement vectors was shown to be strictly dependent on the core protein of the HR pathway, RAD51 (Schaefer *et al*., 2010; Wendeler *et al*., 2015). Therefore, we assessed its implication in CRISPR‐Cas9‐induced GT. This experiment showed that GT efficiencies in the absence of PpRAD51 were still possible but were reduced to ca. 30% of those observed in the WT (Table 5). A strong rise of the contribution of IR reactions in transgene integration is consistent with our previous characterisation of *Pprad51* mutants (Schaefer *et al*., 2010). Yet, HDR‐mediated GT in the absence of PpRAD51 is a surprising result and uncovers an alternative HDR‐mediated GT pathway that seems to be only active following CRISPR‐Cas9‐induced GT. Further experiments are needed to identify the mechanisms leading to homology‐mediated integration of a donor template without RAD51 proteins. One candidate pathway could be the well‐described RAD52‐dependant SSA pathway (Ceccaldi *et al*., 2016; Symington, 2002), and we are currently investigating this possibility. Indeed, RAD52 is essential for HDR‐mediated targeted integrations and loss of RAD51 function hardly affects GT efficiency in *S. cerevisiae* (Schiestl *et al*., 1994).

In this report, we have shown that the use of CRISPR‐Cas9 allows efficient targeted mutagenesis and significantly improves GT efficiency and precision in *P. patens*, expanding the range of available tools for gene function analysis in this model organism and facilitating the production of moss‐made pharmaceutical, a very promising new area of biotechnology (Reski *et al*., 2015). Finally, our work also uncovers novel features of CRISPR‐induced HDR‐mediated GT that could lead to improve the efficiency of such approach in GT noncompetent cells.

## Experimental procedures

### Protoplast isolation and transformation

Tissues from the Gransden wild‐type strain of *Physcomitrella patens* (Ashton and Cove, 1977) and from the Pp*rad51‐1‐2* double mutant (Schaefer *et al*., 2010) were propagated and protoplasts isolated and transformed as previously described (Schaefer and Zrÿd, 1997). Plasmid DNA was extracted with the Nucleobond XA kit (Macherey‐Nagel, France). Protoplasts were transformed with 10 to 25 μg circular DNA and then spread on a regeneration medium composed of PpNO3 medium (Ashton *et al*., 1979), supplemented with 2.7 mm NH4‐tartrate (PpNH4 medium) and 0.33 m mannitol for a week before selection.

### Molecular cloning

The pAct‐Cas9 plasmid used in this study contains a Cas9 expression cassette containing the rice actin 1 promoter and a codon‐optimized version of Cas9 from *Streptococcus pyogenes* fused to a SV40 nuclear localization (Mali *et al*., 2013). The pAct‐Cas9 plasmid was constructed as follows: the hCas9 plasmid (plasmid#41815 from AddGene) was digested by *Nco*I and *Pme*I and the hCas9 gene was ligated to the pCOR104‐CaMVter plasmid (Proust *et al*., 2011) previously digested by *Nco*I and *Sma*I.

Two sgRNA expression cassettes were designed, each containing a U6 promoter from *P. patens*, the 5′‐G‐N_(19)_‐3′ sequences targeting *PpAPT* and the tracrRNA scaffold (Mali *et al*., 2013; Figures 1 and S1). *P. patens* genomic sequence for the U6 gene (coordinates 5050300–5050958 on chromosome 1) was identified by Basic Local Alignment Search Tool (http://www.phytozome.net/physcomitrella_er.php) using the Arabidopsis U6‐26 snRNA sequence (X52528; Li *et al*., 2007) as query. U6 promoter sequence coordinates used for gRNA expression are 5050300–5050621 on chromosome 1. For the design of CRISPR‐Cas targets in the *PpAPT* gene, both strands of the *P. patens* adenine phosphoribosyltransferase gene (PpAPT, Phytozome # Pp3c8_16590) were searched using the CRISPOR, free software (http://tefor.net/crispor/crispor.cgi), for sequences of the form 5′‐G‐N(18 or 19)NGG‐3′ with respect to the U6 promoter and Cas9 specificity conditions. Two target loci were selected, one in exon 5 (sgRNA#1) and one in exon 3 (sgRNA#2) of the *PpAPT* gene (Figure 1). The sgRNA1 and sgRNA2 cassettes were synthesized as gBlocks^®^ by IDT (www.idtdna.com), PCR‐amplified and introduced into pCR^*®*^ *II‐*TOPO^®^ TA‐cloning vectors (www.lifetechnologies.com) to give the plasmids psgRNA#1 and psgRNA#2.

Two donor DNA cassettes were used for gene targeting experiments. The PpAPT‐KO4 knockout cassette used for gene targeting experiments bears a 715‐bp 5′ targeting fragment (coordinate 772–1486 on Pp3c8_16590 in Phytozome) and a 702‐bp 3′ targeting fragment (coordinate 1487–2188 on Pp3c8_16590 in Phytozome) of the *PpAPT* gene, flanking a pAct :: hygroR cassette from the pActHygR plasmid. The pActHygR carries a *HPH* gene for resistance to hygromycin (Bilang *et al*., 1991) in fusion with the rice actin 1 promoter from pCOR104 (McElroy *et al*., 1991) and before a NOS terminator. The 5′ and 3′ sequences of the *PpAPT* gene present in the PpAPT‐KO4 cassette are flanking the predicted CRISPR‐mediated DSB for target#1 sequence (coordinate 1468–1487 on Pp3c8_16590 in Phytozome). The PpAPT‐KO7 knockout cassette bears a 743‐bp 5′ targeting fragment (coordinate 156–898 on Pp3c8_16590 in Phytozome) and a 778‐bp 3′ targeting fragment (coordinate 917–1694 on Pp3c8_16590 in Phytozome) of the *PpAPT* gene, flanking a 35S :: neoR cassette from pBNRF for resistance to G418 (Schaefer *et al*., 2010) cloned in a pCR^*®*^ *II‐*TOPO^®^ TA‐cloning vector. The 5′ and 3′ sequences of the *PpAPT* gene present in the PpAPT‐KO7 cassette are flanking the predicted CRISPR‐mediated DSB for the target#2 sequence (coordinate 890–909 on Pp3c8_16590 in Phytozome).

### Gene knockout assays

Moss protoplasts (4.8 × 10^5^) were cotransformed with the pAct‐Cas9 and psgRNA#1 or psgRNA#2 plasmids. Non‐sense mutations in the *PpAPT* gene confer resistance to the toxic adenine analogue 2‐fluoroadenine (2‐FA). Regenerating protoplasts were selected on PpNH4 supplemented with 10 μm 2‐FA (Fluorochem) to detect clones which had been disrupted at the *PpAPT* locus. Experiments were repeated three times.

For the characterisation of the mutations triggered in the *PpAPT* gene by the sgRNA/Cas9 system, two sets of primers surrounding the target sites were designed. For target#1, we used the primers PpAPT#15 and PpAPT#19. For target#2, we used the primers PpAPT#5 and PpAPT#14. To check the specificity of our two sgRNAs, we designed primers surrounding all the potential off‐target sites identified with the CRISPOR software (Figure S4).

### Gene targeting assays

For the CRISPR‐Cas9‐mediated gene targeting experiment, moss protoplasts (4.8 × 10^5^) were cotransformed with the pAct‐Cas9, psgRNA#1 and PpAPT‐KO4 plasmids or with the pAct‐Cas9, psgRNA#2 and PpAPT‐KO7 plasmids. For the ‘classical’ gene targeting experiment, moss protoplasts (4.8 × 10^5^) were transformed with the PpAPT‐KO4 plasmid digested with *BsaA*I and *Hind*III or with the PpAPT‐KO7 plasmid digested with *BsmB*I and *BstX*I. Targeted integration of the PpAPT‐KO4 or PpAPT‐KO7 cassettes at the *PpAPT* gene confers resistance to 2‐FA. We selected primary transformants (unstable + stable) with 50 mg/L G418 (Duchefa). Integrative transformants were isolated following a second round of selection on G418. Small pieces of protonema tissue from these transformants were then transferred onto PpNH4 medium containing 10 μm of 2‐FA to detect *PpAPT* gene targeting events. Experiments were repeated three times. GT efficiencies were determined as the frequency of 2‐FA‐resistant plants among antibiotic‐resistant transformants (targeted + random insertion of the donor DNA template).

For analysis of the nature of the HDR event, that is targeted gene replacement (TGR) versus targeted gene insertion (TGI; Kamisugi *et al*., 2006), the antibiotic‐resistant clones that were also 2‐FA resistant were genotyped. The molecular analysis of the left and right junctions of the insertions was performed using primers specific to the PpAPT‐KO4 or PpAPT‐KO7 cassettes and primers located in the *PpAPT* gene but outside of the genomic fragments present on the donor DNA cassettes. The 5′ junction was detected using the primers PpAPT#8 and 35SProRev#3, and the 3′ junction using PpAPT#5 and 35STerFwd#3. The number of inserted copies of the donor DNA template at the target site was estimated using a set of primers located outside of the genomic fragments present in the knockout cassette, PpAPT#5 and PpAPT#8, which amplified fragments of 1700 bp for wild‐type clones and fragments of 3700 bp for monocopy insertions. Multiple insertions of the cassette led to no amplification in our conditions.

### DNA DSB repair pathway choice assays

For the characterisation of the nature of DNA DSB repair after CRISPR‐Cas9‐mediated DSB in presence of a donor DNA template, moss protoplasts (4.8 × 10^5^) were cotransformed with the pAct‐Cas9, psgRNA#2 and PpAPT‐KO7 plasmids. Transformants where the *PpAPT* gene has been disrupted due to non‐sense mutations (via IR) or to targeted integration (via HDR) were selected on PpNH4 supplemented with 10 μm 2‐FA for 2 weeks. Small pieces of protonema tissue from these transformants were then transferred onto PpNH4 medium containing 50 mg/L G418 (Duchefa) to detect *PpAPT* gene targeting events. Experiments were repeated three times. Proportion of the DSBs that were repaired via HDR was determined as the rate of G418‐resistant plants among 2‐FA‐resistant transformants.

### PCR analysis of the transformants

For PCR analysis, genomic DNA was extracted from 50 mg of fresh tissue using a genomic DNA quick preparation procedure previously described (Trouiller *et al*., 2006). For the sequences of the PCR primers used in this study, see Figures S5–S7. The quality of the DNA samples was controlled using primers targeting the *SGS1* gene from *P. patens*: sgs1‐Fwd#7 and sgs1‐Rev#8.

## Supporting information


**Figure S1** Schematic description of the sgRNA/Sp‐hCas9 system.
**Figure S2** Effect of relative concentrations of Cas9 and sgRNAs on the efficiency of the CRISPR‐Cas9 system in *P. patens*.
**Figure S3** Hypothesis on the DNA repair mechanisms explaining frequent deletions observed in the target sequences.
**Figure S4** Sequences and positions of possible off target sites for sgRNA1 and sgRNA2.
**Figure S5** Sequences of primers used.
**Figure S6** Genotyping of the clones selected in the CRISPR‐induced gene targeting experiments using sgRNA#2 and PpAPT‐KO7 donor cassette.
**Figure S7** Detection of single copy insertion of the donor cassette at the target site.
**Figure S8** Genotyping of clones selected in the CRISPR‐induced gene targeting experiments using sgRNA#1 and PpAPT‐KO4 donor cassette in the wild type and in the double mutant *Pprad51‐1‐2*.
