# Supplementary material for: CRISPR‐Cas9‐mediated efficient directed mutagenesis and RAD51‐dependent and RAD51‐independent gene targeting in the moss Physcomitrella patens
Source: Plant Biotechnol J. 2016 Jul 22;15(1):122–31. doi: 10.1111/pbi.12596 (PMC5253467; doi:10.1111/pbi.12596)
Supplement: Supplementary file 1 — Figure S1 Schematic description of the sgRNA/Sp‐hCas9 system. Figure S2 Effect of relative concentrations of Cas9 and sgRNAs on the efficiency of the CRISPR‐Cas9 system in P. patens. Figure S3 Hypothesis on the DNA repair mechanisms explaining frequent deletions observed in the target sequences. Figure S4 Sequences and positions of possible off target sites for sgRNA1 and sgRNA2. Figure S5 Sequences of primers used. Figure S6 Genotyping of the clones selected in the CRISPR‐induced gene targeting experiments using sgRNA#2 and PpAPT‐KO7 donor cassette. Figure S7 Detection of single copy insertion of the donor cassette at the target site. Figure S8 Genotyping of clones selected in the CRISPR‐induced gene targeting experiments using sgRNA#1 and PpAPT‐KO4 donor cassette in the wild type and in the double mutant Pprad51‐1‐2. [file PBI-15-122-s001.docx]

(A)

5’-G**NNNNNNNNNNNNNNNNNNN**

CNNNNNNNNNNNNNNNNNNNNCC

GNNNNNNNNNNNNNNNNNNNNGG

N

NNNNNNNN-3’

NNNNNNNN-5’

N

5’-NNNNNNNN

N

3’-NNNNNNNN

N

N

N

N

N


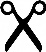

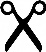


AAGGCUAGUCCGUUAUCAA

UUUU-3’

GUUUUAGAGCUA

UAAAAUU CGAU

GAA

CUUGAAAAAGUGGCACCGA

CGUGGCU

G

GAAA

.

.

(B)

PpU6 promoter + sgRNA#1

ATTGAATGTCCATTGAAGCAGACGTGTTGCGACAGGTTAGCGACGATGGG

TGTAGATGTGATGTGATGTGATGGTGTGGTTCTTCCACGGCGGCGTCCTT

GCGGTGGCGGAGAAGGGGATATCCCGAAGGAGCGGCAGCGGGAGAGCACA

AGCAGAAAGGGTGCAGTGAGTGAGTGGGTCCAGCTGGGTGGCTGGCCGAG

TGGACGCGACCGGGTTTCGAGGGGGCGGGGGAGAAAAGGGATGGAGCGAG

GGATATAACCCACATGGAATGGAGGTGGGTGTGAAGGCGGGTATATAGGA

AGGTGGAGGACTTACAACCGAAGAGTATAGTCTAGAGTAGTTTTAGAGCT

AGAAATAGCAAGTTAAAATAAGGCTAGTCCGTTATCAACTTGAAAAAGTG

GCACCGAGTCGGTGCTTTT

PpU6 promoter + sgRNA#2

ATTGAATGTCCATTGAAGCAGACGTGTTGCGACAGGTTAGCGACGATGGG

TGTAGATGTGATGTGATGTGATGGTGTGGTTCTTCCACGGCGGCGTCCTT

GCGGTGGCGGAGAAGGGGATATCCCGAAGGAGCGGCAGCGGGAGAGCACA

AGCAGAAAGGGTGCAGTGAGTGAGTGGGTCCAGCTGGGTGGCTGGCCGAG

TGGACGCGACCGGGTTTCGAGGGGGCGGGGGAGAAAAGGGATGGAGCGAG

GGATATAACCCACATGGAATGGAGGTGGGTGTGAAGGCGGGTATATAGGA

AGGTGGAGGACTTACAACCGGAGCGTTACCGGGACCAGAGTTTTAGAGCT

AGAAATAGCAAGTTAAAATAAGGCTAGTCCGTTATCAACTTGAAAAAGTG

GCACCGAGTCGGTGCTTTT

**Supplemental figure 1. Schematic description of the sgRNA/Sp-hCas9 system.**

(A) Hybridization of sgRNA at target site triggering endonuclease activity of the Sp-hCas9 protein. (B) Sequences of the sgRNA constructs (U6 promoter from *P. patens* is indicated in orange, the protospacers are in green, the tracRNA is in blue).

1 µg

24 µg

0.1 µg

24.9 µg

12.5 µg

12.5µg

24 µg

1 µg

sgRNA#1

hCas9

24.9 µg

0.1 µg

**Supplemental Figure 2. Effect of relative concentrations of Cas9 and sgRNAs on the efficiency of the CRISPR-Cas9 system in *P. patens*.**

One of the deletions observed (12 bp) (x8)

TAGGCCCTGTGATTAGGGAAGAGTA**---------**---TGGTACCGATTGCATTGAGA

Target sequence of sgRNA#1 in genomic DNA

TAGGCCCTGTGATTAGGGAAGAGTATAGTCTAGAGTATGGTACCGATTGCATTGAGA

**(A)**

**Potential scenarios explaining this deletion :**

GGGAAGAGTATGGTACCGAT

CCCTTCTCATACCATGGCTA

GGGAAGAgtatagtctagaGTATGGTACCGAT

CCCTTCTcatatcagatctCATACCATGGCTA

sgRNA#1/Cas9

GGGAAGA------------GTATGGTACCGAT

CCCTTCT------------CATACCATGGCTA

Ligase ?

**DSB repair via NHEJ**

GGGAAGAgtatagtctaga**GTA**TGGTACCGAT

CCCTTCT**CAT**ATCTGATCTcatACCATGGCTA

sgRNA#1/Cas9

GGGAAGA**GTA**TGGTACCGAT

CCCTTCT**CAT**ACCATGGCTA

GGGAAGA**GTA**TGGTACCGAT

CCCTTCT**CAT**ACCATGGCTA

ATCTGATCT


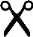


Triming complex ?

**DSB repair via alt-EJ**

One of the deletions observed (12 bp) (x32)

GATCGACATCTTTGTTGAGCGTTACCGGGA**C-----------**-GTCATTGTGG

Target sequence of sgRNA#2 in genomic DNA

GATCGACATCTTTGTTGAGCGTTACCGGGACCAGAAGGTGGACGTCATTGTGG

**(B)**

**Potential scenarios explaining this deletion :**

AGAAGGT

G


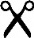

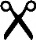


GTTGAGCGTTACCGggaccAGAAGGT**GGAC**GT

CAACTCGCAATGGC**CCTG**GtcttccacctgCA

GTTGAGCGTTACCG**GGAC**GT

CAACTCGCAATGGC**CCTG**CA

GTTGAGCGTTACCG**GGAC**GT

CAACTCGCAATGGC**CCTG**CA

Triming complex ?

sgRNA#2/Cas9

GTTGAGCGTTACCGggaccagaAGGTGGACGT

CAACTCGCAATGGCcctggtctTCCacctgCA

GTTGAGCGTTACCGGGACGT

CAACTCGCAATGGCCCTGCA

GTTGAGCGTTACCG------------GGACGT

CAACTCGCAATGGC------------CCTGCA

sgRNA#2/Cas9

Ligase ?

**DSB repair via NHEJ**

**DSB repair via alt-EJ**

**Supplemental Figure 3**. **Hypothesis on the DNA repair mechanisms explaining frequent deletions observed in the target sequences.**

Some of the deletions frequently observed in the sequences targeted by sgRNA#1 (A) and sgRNA#2 (B) could be due either to alt-EJ or NHEJ-directed repair. In green the target of sgRNA#1 (A) and sgRNA#2 (B), in red the PAM. In small letters, the ressected sequences on both sides of the CRISPR/Cas-induced cleavage. In bold letters, the sequences potentially hybridizing to generate by alt-EJ the observed deletions after the overlap DNA has been cut-off. (The frames refer to the micro-homologies mentioned in Figure 2.)

| **Locus name** | **Target sequence + PAM^a^** | **Position on *P. patens* reference genome from Phytozome^a^** |
| --- | --- | --- |
| **sgRNA1 Target** | **gaagagtatagtctagagtaTGG** | Chr08:10812103 to 10812081 |
| sgRNA1 Off-Target#1 | gaagagtgtagagtagagtaGGG | Chr07:4705222 to 4705244 |
| sgRNA1 Off-Target#2 | gaagcttattgtctagagttAGG | Chr05:17438841 to 17438819 |
| sgRNA1 Off-Target#3 | gtaaagtatagtctagggttGGG | Chr24:6867383 to 6867405 |
| sgRNA1 Off-Target#4 | caagcgtatactcttgagtaTGG | Chr14:2675353 to 2675375 |
| sgRNA1 Off-Target#5 | gaatattataatctagagttGGG | Chr22:3453377 to 3453399 |
| sgRNA1 Off-Target#6 | gcaaagtatagtataaagtaGGG | Chr06:582960 to 582982 |
| sgRNA1 Off-Target#7 | gaagactatagtttagagggCGG | Chr02:13705150 to 13705128 |
| sgRNA1 Off-Target#8 | gaaaagtattgtcaagactaAGG | Chr05:6043528 to 6043550 |
| sgRNA1 Off-Target#9 | gaagactatattctatactaCGG | Chr17:5887705 to 5887683 |
| **sgRNA2 Target** | **tgagcgttaccgggaccagaAGG** | Chr08:10812681 to 10812659 |
| sgRNA2 Off-Target#1 | tgaĝgagtccaagggaccagaAGG | Chr16:7631517 to 7631494 |
| sgRNA2 Off-Target#2 | tgaaggttacctggaccag=AGG | Chr4:12028646 to 12028667 |
| sgRNA2 Off-Target#3 | tgggcgtgacggggagcagaAGG | Chr17:4446370 to 4446348 |
| sgRNA2 Off-Target#4 | tgagcgtttcaggtacctgaAGG | Chr09:9115552 to 9115574 |

^a^ Coordinates of the off target sequences were identified using Basic Local Alignment Search Tool (http://phytozome.jgi.doe.gov/pz/portal.html#!search).

**Supplemental Figure 4: Sequences and positions of possible off target sites for sgRNA1 and sgRNA2**.

Mismatches with the target sites are indicated in red. *P. patens* genomic off target sequences were identified using the CRISPOR v2.0 tool (http://crispor.tefor.net).

| **Primers** | **Sequences** |
| --- | --- |
| PpSGS1#7 | GATAGACTGTCGCAGCATCTTAC |
| PpSGS1#8 | TGATTTGCACTTCGCTATAACT |
| PpAPT#2 | TTTTTGCGCTCGCTGTTTCTG |
| PpAPT#5 | ACAAGGTGGTGTCAACTTTCAAGG |
| PpAPT#8 | AACGCGAGGGTGACCCAAGCC |
| PpAPT#14 | ATGTTTCAGTCCTTCGTTTG |
| PpAPT#15 | TATGTTCCGAGATGTGACGAC |
| PpAPT#19 | CCCGACAACTTCTCACGACCC |
| PpAPT#20 | TAAATAATTCTGACCCAAAGT |
| 35SProRev#3 | GTCTTGCGAAGGATAGTGGG |
| 35STerFwd#3 | CGCTGAAATCACCAGTCTCTCT |
| ActProRev#1 | CGGACAACTCACGGTGATAGTG |
| NosTer#1 | CATCTCGAGTAGCCGCGGGAATTTCCCCG |
| gRNA1OT1#1 | GCTGATAAGCGTGGTGCAGA |
| gRNA1OT1#2 | TGGGGAGGGTGGATACAGAA |
| gRNA1OT2#1 | AGAAGTTGGCATATTGAGGTCGT |
| gRNA1OT2#2 | GAGAATGCACCGAGCGACTA |
| gRNA1OT3#1 | GCAGCAAGTGCTGGTTCTTC |
| gRNA1OT3#2 | AGCGCGCAGTCTCTGTTTAT |
| gRNA1OT4#1 | AGCTCCTTTGAAGCCTTACCA |
| gRNA1OT4#2 | ACGAGCCTAGACTATGTGAGA |
| gRNA1OT5#3 | GGCAAGTTGCAAGTGGTCTG |
| gRNA1OT5#4 | CGCAGGTGAGATTTGGGACT |
| gRNA1OT6#1 | GTCGGGGTTGATCCGATTGA |
| gRNA1OT6#2 | CTCTCTTTTCCCTGCCTCCG |
| gRNA1OT7#1 | CTTGCAGGCCGTGATAATGC |
| gRNA1OT7#2 | TCCGCATCTTGGTGTGTGAA |
| gRNA1OT8#2 | TCTCTTCGTAGAGCTCGGAGT |
| gRNA1OT8#3 | TTGTTTGAAAGATCTTATATCATAG |
| gRNA1OT9#2 | TCATGTTTGTCCATGTTTTTGGA |
| gRNA1OT9#3 | TGTTGGCAAAATGCCTACCT |
| gRNA2OT1#1 | GCAGAGGAACAGAAGGAACC |
| gRNA2OT1#2 | GAGCGTCATCTGCTTGCTTG |
| gRNA2OT2#1 | TCGGTTTTGCAGCTGCTTTC |
| gRNA2OT2#2 | GAGCAAAGCACAGTATGCGG |
| gRNA2OT3#1 | CTCTTTCCCCGCATTCCTGT |
| gRNA2OT3#2 | CTACGGAGCCGTTCACCATT |
| gRNA2OT4#1 | TCTTCACAGCAGGACGAACC |
| gRNA2OT4#2 | AGCAGAACAAGCTCCTCGAC |

**Supplemental Figure 5: Sequences of primers used.**

TGR: Targeted Gene Replacement

TGI: Targeted Gene Insertion

3kb

1kb

5’ junction: PpAPT#8 + 35SProRev#3 (1.5kb)

1kbL 1 2 3 4 5 6 7 8 9 10


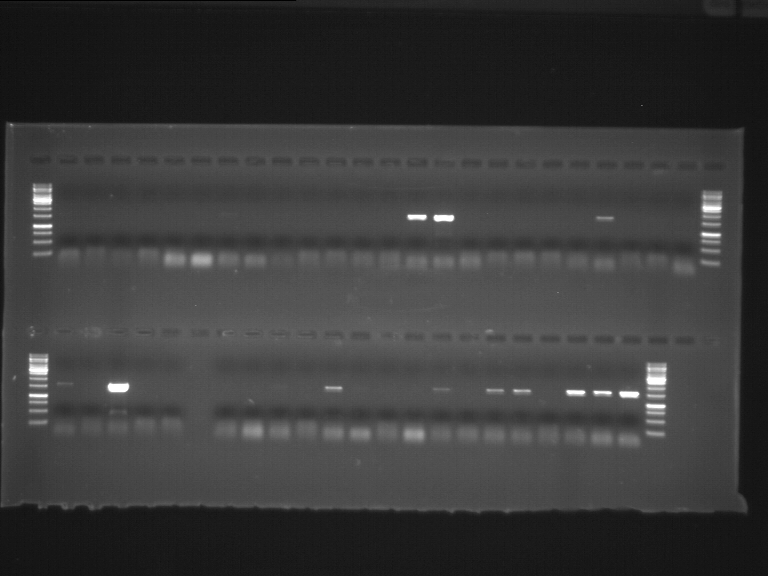


3kb

1kb

3’ junction: PpAPT#5 + 35STerFwd#3 (1.2kb)

1kbL 1 2 3 4 5 6 7 8 9 10


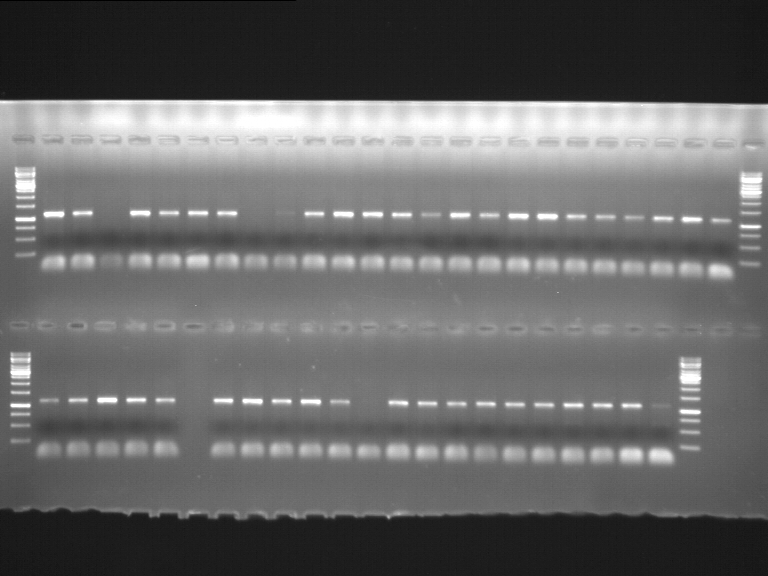


Interpretation

1 2 3 4 5 6 7 8 9 10

5’ junction + + + - + + - + - -

3’ junction + + + + + + + + + +

DNA control + + + + + + + + + +

HDR event TGR TGR TGR TGI TGR TGR TGI TGR TGI TGI

3kb

1kb

DNA control: PpSGS1#7 + PpSGS1#8 (0.7kb)

1kbL 1 2 3 4 5 6 7 8 9 10


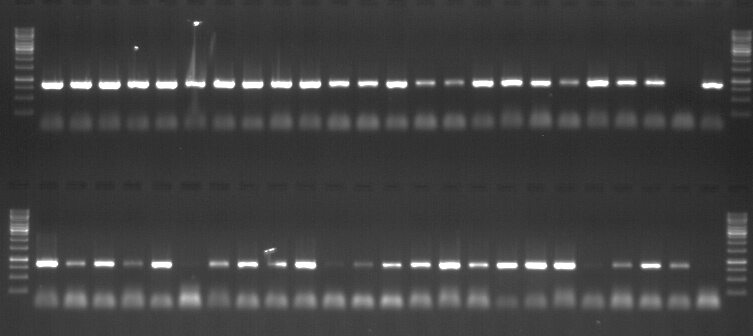


**Supplemental Figure 6**: **Genotyping of the clones selected in the CRISPR-induced gene targeting experiments using sgRNA#2 and PpAPT-KO7 donor cassette.**

Example of genotyping of 2-FA^R^ clones (a total of 95 and 52 clones were analysed for the "CRISPR-Cas9" and "classical" methods of transformation respectively). The 5’ and 3’ junctions of the integration were detected by using primers PpAPT#8 + 35SProRev#3 and PpAPT#5 + 35STerFwd#3 respectively. The *PpSGS1* gene has been used as positive control using primers PpSGS#7 + PpSGS#8. Position of the PCR primers used for genotyping can be found in Figure 1.

3kb

1kb

1kbL 1 2 3 4 5 6 7 8 9 10


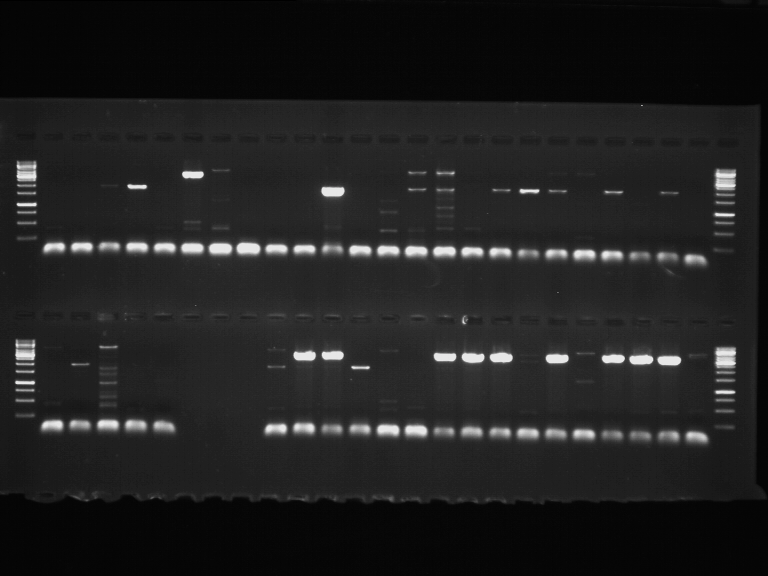


Single copy insertion: PpAPT#8+ PpAPT#5 (3.7kb)

1 2 3 4 5 6 7 8 9 10

Single copy - + + + - + - + + +

**Supplemental Figure 7: Detection of single copy insertion of the donor cassette at the target site.**

Example of genotyping of TGR events found previously (a total of 84 and 40 clones were analysed for the "CRISPR-Cas9" and "classical" methods of transformation respectively, see Supplemental Figure 5). The primers used (PpAPT#8 and PpAPT#5) are located outside the genomic fragments present in the cassette (Figure 1B).

3kb

1kb


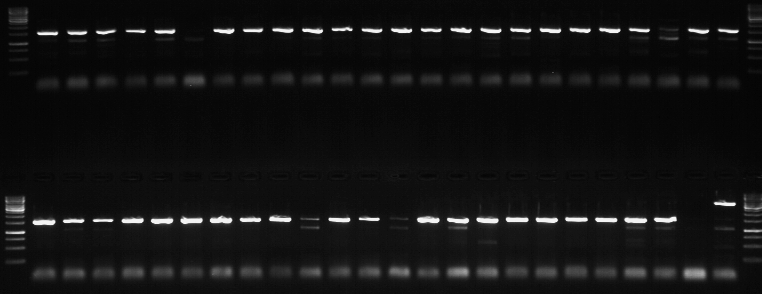

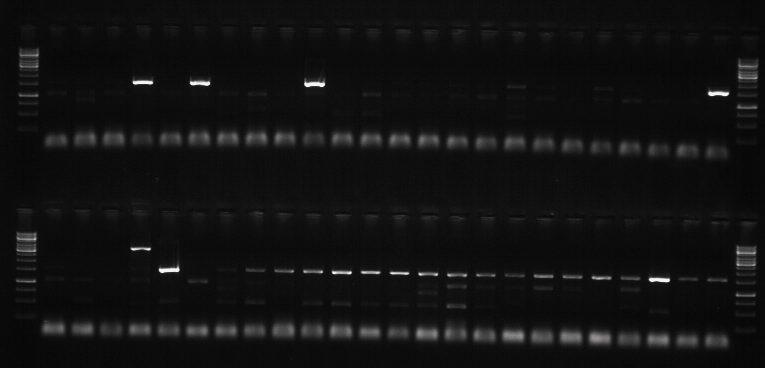


1kbL 1 2 3 4 5 1kbL 1 2 3 4 5

5’ junction: PpAPT#2+ ActProRev#1 (2kb)

3kb

1kb


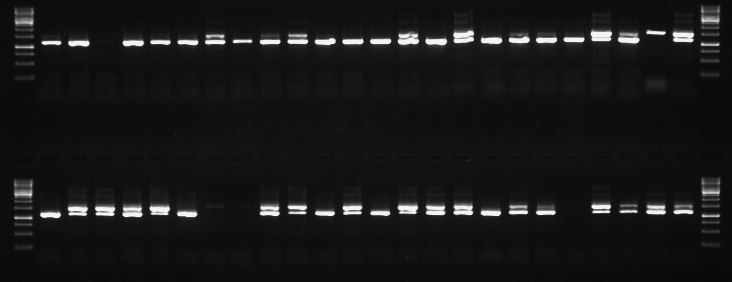

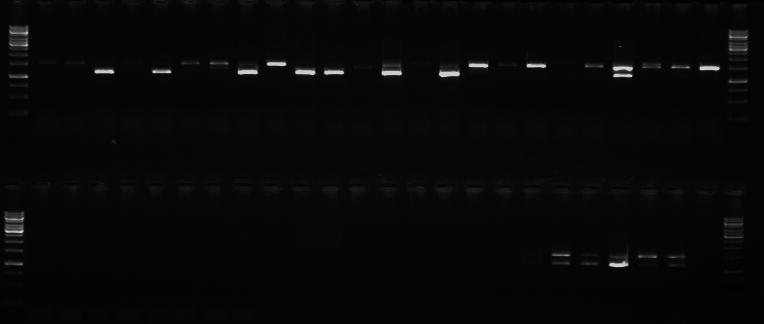


1kbL 1 2 3 4 5 1kbL 1 2 3 4 5

3’ junction: PpAPT#20 + NostTer#1(1.2kb)

3kb

1kb


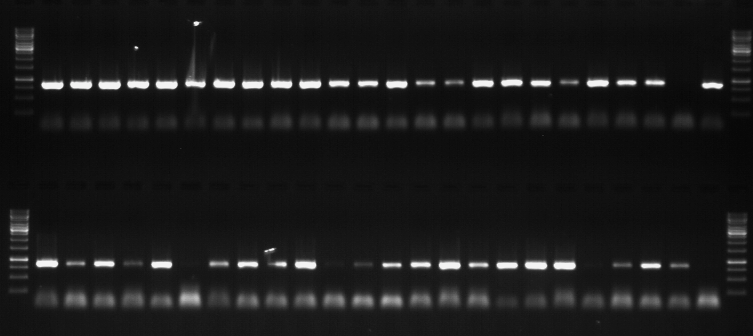

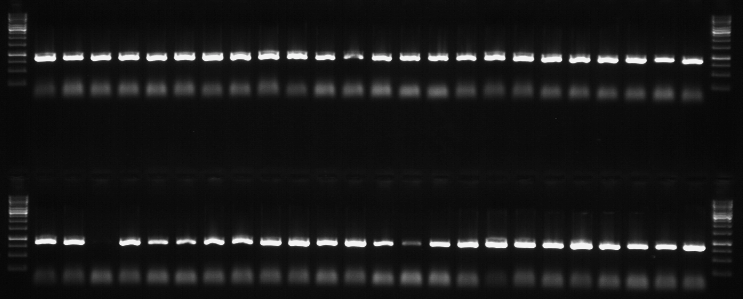


1kbL 1 2 3 4 5 1kbL 1 2 3 4 5

DNA control: PpSGS1#7 + PpSGS1#8 (0.7kb)

3kb

1kb

1kbL 1 2 3 4 5 1kbL 1 2 3 4 5

APT locus: PpAPT#5 + PpAPT#14 (1.2kb)


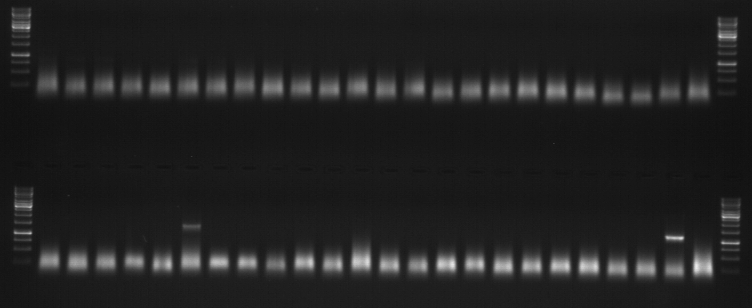

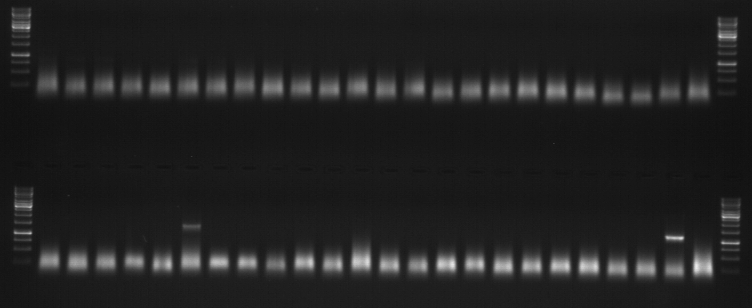


1 2 3 4 5 1 2 3 4 5

5’ junction + + + + + - - - + -

3’ junction + + - + + - - + - +

APT locus - - - - - - + - - -

DNA control + + + + + + + + + +

Event TGR TGR TGI TGR TGR IR RI TGI TGI TGI

WT *Pprad51-1-2*

TGR: Targeted Gene Replacement

TGI: Targeted Gene Insertion

IR: Illegitimate Recombination

RI: Random Integration

WT *Pprad51-1-2*

WT *Pprad51-1-2*

WT *Pprad51-1-2*

WT *Pprad51-1-2*

**Supplemental Figure 8**: **Genotyping of clones selected in the CRISPR-induced gene targeting experiments using sgRNA#1 and PpAPT-KO4 donor cassette in the wild type and in the double mutant *Pprad51-1-2*.**

Example of genotyping of 2-FA^R^ clones (a total of 47 and 93 clones were analysed for the WT and the *Pprad51-1-2* mutant respectively). The 5’ and 3’ junction fragments were detected by using primers PpAPT#2+ ActProRev#1 and PpAPT#20 + NosTer#1 respectively. The *PpAPT* locus was amplified using PpAPT#5+PpAPT#14. The *PpSGS1* gene has been used as positive control using primers PpSGS#7 + PpSGS#8. Position of the PCR primers used for genotyping can be found in Figure 1.
